# Metagenome-Assembled Genome of a *Pseudanabaena* sp. from a Crimson Cyanobacterial Bloom in Lake Salubria, New York, USA

**DOI:** 10.1128/mra.00494-22

**Published:** 2022-08-16

**Authors:** Robbie M. Martin, Eric R. Gann, Alex R. Truchon, Gregory L. Boyer, Steven W. Wilhelm

**Affiliations:** a Department of Microbiology, University of Tennessee, Knoxville, Tennessee, USA; b Department of Chemistry, State University of New York College of Environmental Science and Forestry, Syracuse, New York, USA; Montana State University

## Abstract

*Pseudanabaena* spp. are filamentous cyanobacteria widely distributed in temperate lakes. Though infrequent, they can form harmful algal blooms. Here, we present a high-quality metagenome-assembled genome of a *Pseudanabaena* sp. from a toxic, crimson cyanobacterial bloom in Lake Salubria, NY.

## ANNOUNCEMENT

*Pseudanabaena* spp. are filamentous, nonheterocystous cyanobacteria widely distributed in temperate lakes (1). *Pseudanabaena* spp. can form harmful algal blooms (2) and are difficult to distinguish from the morphologically similar *Limnothrix* spp., with which they cluster in phylogenies based on 16S rRNA (3). Here, we present a metagenome-assembled genome (MAG) of *Pseudanabaena* sp. Salubria-1, collected from Lake Salubria, NY, USA.

**FIG 1:**
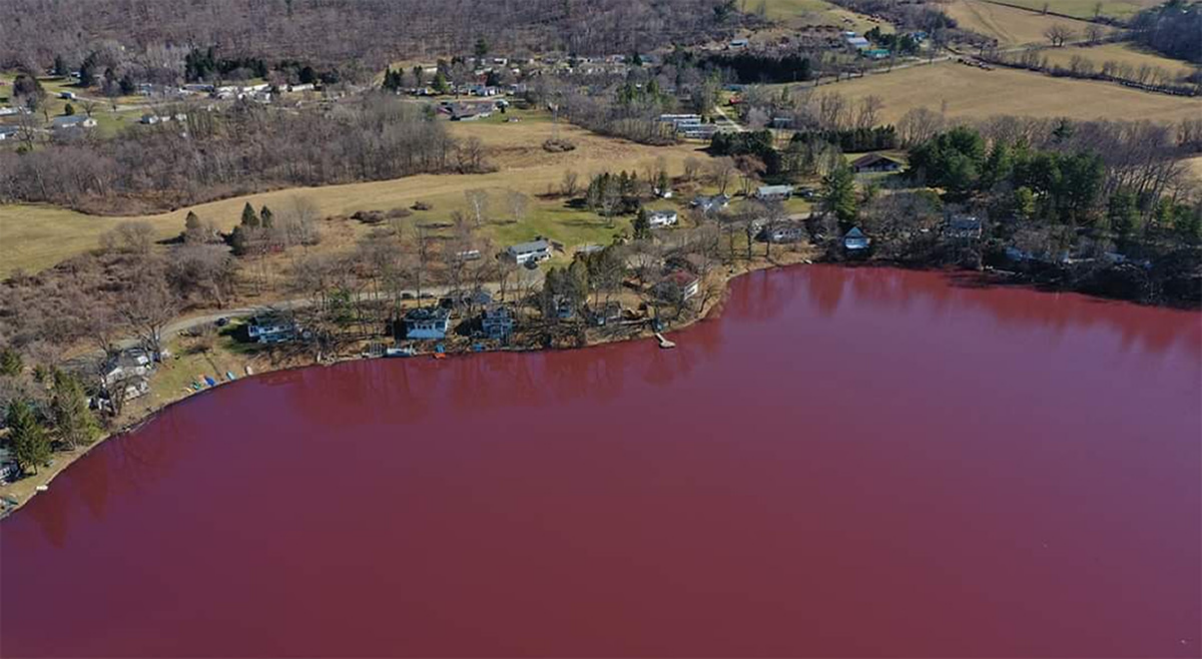
Aerial view of Lake Salubria, NY, USA, in spring 2020 illustrating a crimson color caused by a cyanobacterial bloom. Photo credit: Dillon Lewis, Lewis Imagery, Bath, NY.

Lake Salubria is an ~24-ha kettle hole lake in the Finger Lakes region near Bath, NY, USA (42.329°N, 77.293°W). In spring 2020, an algal bloom turned the lake a vivid crimson, concerning the community (Fig. 1). Grab samples of the bloom were collected 1 April 2020, when methyl-microcystin-LR concentrations ranged from 2 to 4 μg L^−1^. Enrichment cultures were grown in MLA medium (4) at 19°C and ~30 μmol photon m^−2^ s^−1^ on a 12-h:12-h light/dark cycle. Crimson filaments dominated the enrichment cultures. We sequenced enrichment culture samples to identify the causative organism and identify potential toxin producers.

Filaments were filtered onto a 5-μm polycarbonate filter from which genomic DNA was extracted using standard phenol-chloroform methods (5). Short-read libraries were prepared and sequenced by the Microbial Genome Sequencing Center (Pittsburgh, PA) using the Illumina Nextera kit and the Illumina NextSeq 550 platform, which generated 8,496,556 151-bp paired-end reads. Long-read libraries were prepared using Oxford Nanopore ligation sequencing kit SQK-LSK1 and sequenced in-house on a Nanopore MinION Mk1B using an R9.4.1 flow cell. DNA originated from the same extraction as used for short reads and was neither sheared nor size selected. Nanopore sequencing generated 260,242 reads (*N*_50_ = 5,395 bp).

Default parameters were used for all software unless otherwise noted. For Nanopore reads, bases were called using guppy (v4.0.15 + 56940742) (6), adapter trimmed using Porechop (v0.2.4) (7), and trimmed for quality using NanoFilt (v2.7.1) (8). Illumina reads were trimmed in CLC Genomics Workbench (v20.0). *De novo* assembly was conducted using Unicycler (v0.4.9b) in normal mode (9). MAGs were recovered using MaxBin2 (v2.2.4) (10). CheckM (v1.0.18) was used to evaluate MAG quality (11). GTDB-Tk (v1.7.0) was used to assign taxonomy (12). The genome was annotated using the Prokaryotic Genome Annotation Pipeline (PGAP) (v6.1) (13). The 16S rRNA gene sequence used in phylogenetic analysis was extracted from this assembly.

We recovered a MAG of *Pseudanabaena* sp. Salubria-1, consisting of 107 contigs with a total length of 7,138,199 bp (*N*_50_ = 145,348 bp) and a GC content of 41.93%. The sequencing depth was ~191-fold. The genome was estimated at 99.1% complete with 3.3% contamination. The closest taxonomic placement (91.9% average nucleotide identity [ANI]) was to *Pseudanabaena* sp. strain UWO311, a red strain isolated from Dickson Lake, Ontario, Canada (14). This was supported by 16S rRNA gene phylogeny, which placed Salubria-1 next to UWO311 within the *Pseudanabaena/Limnothrix* group (1). Salubria-1 and UWO311 16S rRNA genes shared 99.4% nucleotide identity (1,477/1,486). PGAP predicted 6,982 genes, 6,776 proteins, 54 tRNAs, and 6 rRNAs.

### Data availability.

The metagenome-assembled genome is deposited at DDBJ/ENA/GenBank under accession number JALQCR000000000. Reads are deposited in the NCBI Sequence Read Archive under accession numbers SRX15003418 and SRX15003417.
